# The complete chloroplast genome sequences of *Trapa quadrispinosa* and *T. bicornis* var. *taiwanensis*

**DOI:** 10.1080/23802359.2020.1871433

**Published:** 2021-02-09

**Authors:** Manlin Feng, Jianhua Xue, Jie Zhang

**Affiliations:** a College of Landscape and Architecture, Northeast Forestry University, Harbin, China; b State Key Laboratory of Vegetation and Environmental Change, Institute of Botany, Chinese Academy of Sciences, Beijing, China

**Keywords:** *Trapa*, chloroplast genome, phylogenetic analysis

## Abstract

The complete chloroplast genomes of *Trapa quadrispinosa* and *T. bicornis* var. *taiwanensis* were reported in this study. The chloroplast genome of *T. quadrispinosa* was 155,554 bp in length, containing an LSC of 88,506 bp, an SSC of 18,274 bp, and a pair of IR regions of 24,387 bp each. The chloroplast genome of *T. bicornis* var. *taiwanensis* was 155,543 bp in length, including an LSC of 88,497 bp, an SSC of 18,274 bp, and a pair of IR regions of 24,386 bp each. Both genomes had 112 genes, consisting of 78 protein-coding genes, 30 tRNA genes, and four rRNA genes. The phylogenetic analysis revealed that the family Trapaceae was closely related to the family Sonneratiaceae.

*Trapa* L. is a genus with annual aquatic plants belonged to the family Trapaceae (Wu et al. 1999). The Amur River and Tumen River Basins and Yangtze River Basin are two biodiversity distribution centers of the genus *Trapa* around the world (Xue et al. 2016). *Trapa* has a high-level content of starch, with important medicinal and economic values. However, there still have many questions on the species delimitation and phylogenetic position of *Trapa* (Xue et al. 2017). In this study, the complete chloroplast genomes of *Trapa quadrispinosa* Roxb. and *T. bicornis* var. *taiwanensis* (Nakai) Z. T. Xiong were reported.

The materials of *T. quadrispinosa* and *T. bicornis* var. *taiwanensis* were collected from Liangzi Lake (30°26′N, 114°45′E, Hubei Province, China) and Hongze Lake (33°22′N, 118°42′E, Jiangsu Province, China), respectively. The specimens of them have been kept in Institute of Botany, Chinese Academy of Sciences (T20181006; T20181016). Total genomic DNA was extracted using the modified CTAB method (Doyle and Doyle 1987) and sequenced using the Illumina Hiseq Platform (Illumina Inc., San Diego, CA). Genomes were assembled by GetOrganelle v1.5 (Jin et al. 2018) and annotated using PGA (Qu et al. 2019).

The chloroplast genome of *T. quadrispinosa* (MT941481) was 155,554 bp in length with a GC content of 36.40%, containing a pair of IRs of 24,387 bp each which divide LSC of 88,506 bp and SSC of 18,274 bp. The chloroplast genome of *T. bicornis* var. *taiwanensis* (MT941480) was 155,543 bp in length with an overall GC content of 36.40%, including an LSC of 88,497 bp, an SSC of 18,274 bp, and a pair of IRs of 24,386 bp each. Both genomes had 112 genes, consisting of 78 protein-coding genes, 30 tRNA genes, and four rRNA genes.

To confirm the phylogenetic position of *T. quadrispinosa* and *T. bicornis* var. *taiwanensis* among the Myrtales species, chloroplast genome sequences were aligned using MAFFT (Katoh and Standley 2013), and maximum-likelihood phylogenetic tree was constructed by MEGA 7.0 (Kumar et al. 2016) with 1000 bootstraps. The maximum-likelihood phylogenetic tree based on 22 complete chloroplast genomes, *Magnolia pyramidata* and *M. odoratissima* in the genus of *Magnolia* of Magnoliaceae family was used as outgroup. The results revealed that the family Trapaceae had a close relationship with the family Sonneratiaceae. *T. quadrispinosa* and *T. bicornis* var. *taiwanensis* were closely related to *T. bicornis* and *T. natans* with a low-level of divergence (Figure 1).

**Figure 1.**
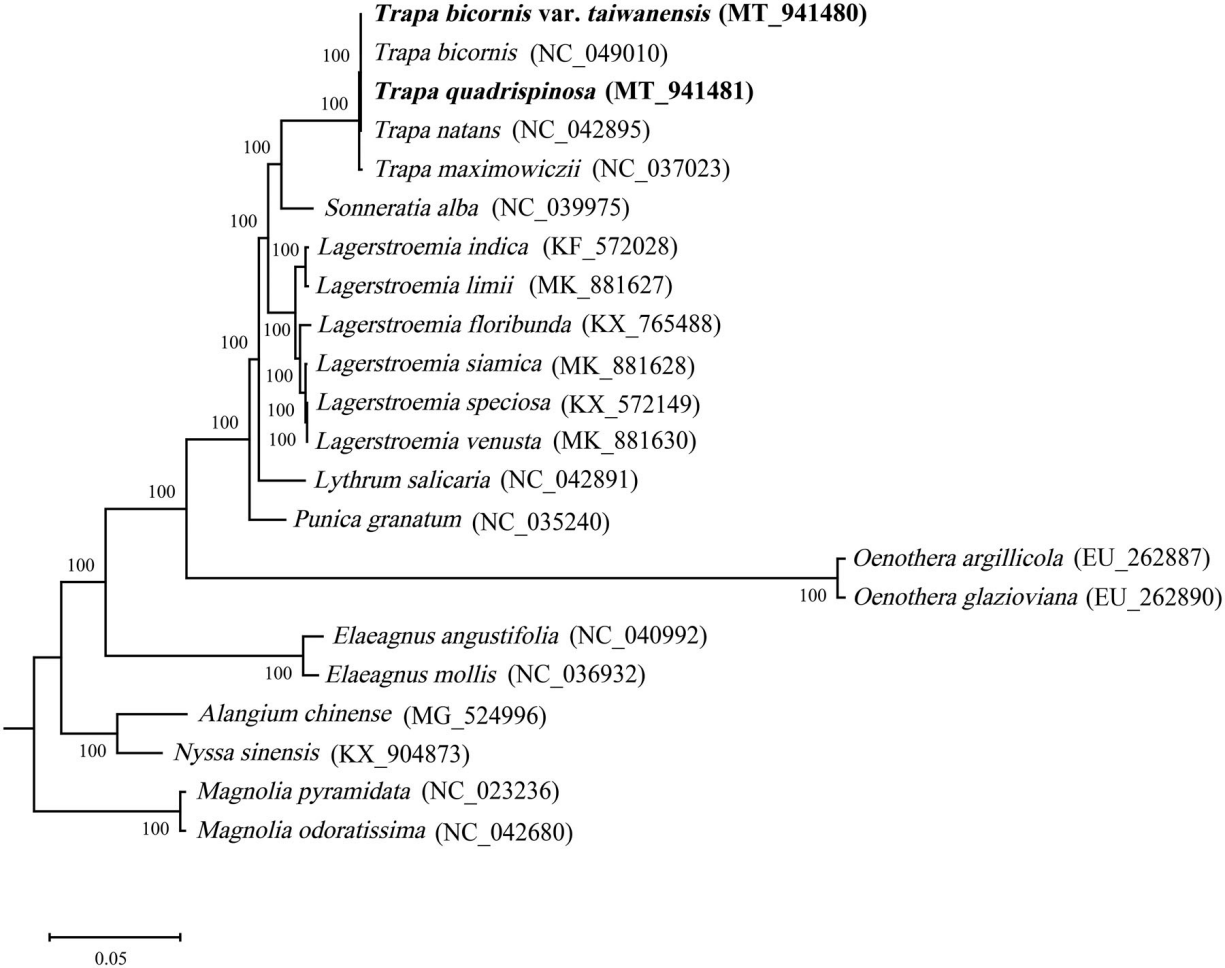
Maximum-likelihood phylogenetic tree based on 22 complete chloroplast genomes with *Magnolia pyramidata* and *M. odoratissima* as outgroup. Numbers on the nodes represent bootstrap values.

## Data Availability

The genome sequence data that support the findings of this study are openly available in GenBank of NCBI at https://www.ncbi.nlm.nih.gov/ under the accession nos. MT941481–MT941480. The associated BioProject, SRA, and Bio-Sample numbers are PRJNA672284, SAMN16562397, and SAMN16562398, respectively.
